# Comparative Analysis of Computer-Aided Diagnosis and Computer-Assisted Subjective Assessment in Thyroid Ultrasound

**DOI:** 10.3390/life11111148

**Published:** 2021-10-27

**Authors:** Nonhlanhla Chambara, Shirley Yuk Wah Liu, Xina Lo, Michael Ying

**Affiliations:** 1 Department of Health Technology and Informatics, The Hong Kong Polytechnic University, Hung Hom, Kowloon, Hong Kong, China; nonhlanhla.chambara@connect.polyu.hk; 2 Department of Surgery, The Chinese University of Hong Kong, Prince of Wales Hospital, Shatin, New Territories, Hong Kong, China; liuyw@surgery.cuhk.edu.hk; 3 Department of Surgery, North District Hospital, Sheung Shui, New Territories, Hong Kong, China; dr.xina@gmail.com

**Keywords:** computer-aided diagnosis, computer-assisted, thyroid nodule, risk-stratification, ultrasound

## Abstract

The value of computer-aided diagnosis (CAD) and computer-assisted techniques equipped with different TIRADS remains ambiguous. Parallel diagnosis performances of computer-assisted subjective assessments and CAD were compared based on AACE, ATA, EU, and KSThR TIRADS. CAD software computed the diagnosis of 162 thyroid nodule sonograms. Two raters (R_1_ and R_2_) independently rated the sonographic features of the nodules using an online risk calculator while blinded to pathology results. Diagnostic efficiency measures were calculated based on the final pathology results. R_1_ had higher diagnostic performance outcomes than CAD with similarities between KSThR (SEN: 90.3% vs. 83.9%, *p* = 0.57; SPEC: 46% vs. 51%, *p* = 0.21; AUROC: 0.76 vs. 0.67, *p* = 0.02), and EU (SEN: 85.5% vs. 79%, *p* = 0.82; SPEC: 62% vs. 55%, *p* = 0.27; AUROC: 0.74 vs. 0.67, *p* = 0.06). Similarly, R_2_ had higher AUROC and specificity but lower sensitivity than CAD (KSThR-AUROC: 0.74 vs. 0.67, *p* = 0.13; SPEC: 61% vs. 46%, *p* = 0.02 and SEN: 75.8% vs. 83.9%, *p* = 0.31, and EU-AUROC: 0.69 vs. 0.67, *p* = 0.57, SPEC: 64% vs. 55%, *p* = 0.19, and SEN: 71% vs. 79%, *p* = 0.51, respectively). CAD had higher sensitivity but lower specificity than both R_1_ and R_2_ with AACE for 114 specified nodules (SEN: 92.5% vs. 88.7%, *p* = 0.50; 92.5% vs. 79.3%, *p* = 0.02, and SPEC: 26.2% vs. 54.1%, *p* = 0.001; 26.2% vs. 62.3%, *p* < 0.001, respectively). All diagnostic performance outcomes were comparable for ATA with 96 specified nodules. Computer-assisted subjective interpretation using KSThR is more ideal for ruling out papillary thyroid carcinomas than CAD. Future larger multi-center and multi-rater prospective studies with a diverse representation of thyroid cancers are necessary to validate these findings.

## 1. Introduction

Ultrasound is the primary imaging modality in the assessment of thyroid nodules. Technological advancements have contributed to the increased use of ultrasound in thyroid cancer diagnosis which has subsequently resulted in overdiagnosis [1,2]. Diverse malignancy risk stratification systems or Thyroid Imaging Reporting and Data Systems (TIRADS) have been developed to improve consistency in subjective interpretation and limit inter-observer variabilities [3,4,5,6]. Nevertheless, variability amongst different TIRADS still exists due to different malignancy risk estimation criteria for suspicious sonographic features [7]. Hence, there is currently no universal standard regarding the best TIRADS to use.

In recent years, an online-based multiple TIRADS malignancy risk scoring calculator based on subjective interpretation of sonographic features was developed [8]. The TIRADS outputs available with this online risk calculator are the American Association of Clinical Endocrinologists/American College of Endocrinology/Associazione Medici Endocrinologi (AACE/ACE/AME—referred to as AACE from hereon), American Thyroid Association (ATA), Korean Society of Thyroid Radiology (KSThR), and French TIRADS. This predictive model-based risk calculator has been evaluated in comparison with other similar subjective interpretation-based models and found to be highly accurate and reliable in thyroid nodule differentiation [9]. On the other hand, with artificial intelligence (AI) evolving, computer-aided diagnosis (CAD) systems have emerged and are suggested as an objective method of thyroid nodule diagnosis. One globally-approved commercial thyroid CAD software with multiple TIRADS computations is AmCAD-UT (AmCad Biomed, Taipei, Taiwan). This CAD software has been evaluated in comparison with human interpreters that were mostly using a single specific TIRADS. Some studies suggested that the CAD software has comparable diagnostic performance to that of experienced clinicians and could potentially improve that of less experienced ones [10,11]. Although both the online risk calculator and the CAD software offer automatic computation of suggested diagnosis based on multiple-TIRADS, presently there is a lack of comparative evaluation of their diagnostic performance for matched multiple TIRADS for clinical adoption considerations. Studies evaluating CAD performance versus that of subjective interpreters have largely focused on the unaided qualitative or quantitative human rating of sonographic features. Moreover, studies on different non-commercialized thyroid CAD technologies have yielded variable results for different TIRADS, thereby leaving the additional value of CAD still ambiguous [12].

The present study aimed to compare the diagnostic performance metrics of computer-assisted subjective analysis by two raters using the online risk calculator, and the commercially available CAD system, based on analogous outputs of different risk-stratification systems. Since CAD software has the potential for screening purposes in low human resource health settings that have no in-house radiologists, the diagnostic performance comparisons were between non-radiologists and CAD in this study. The study sought to construe the clinical application implications of these two computational thyroid nodule diagnosis aides for routine diagnosis adoption considerations in low-resources settings. The hypothesis was that the CAD system has higher diagnostic efficiency than computer-assisted subjective interpretation for all the TIRADS. The rationale is that CAD is suggested to be more objective and less prone to observer bias than subjective interpretation methods. The study findings showed that the sensitivity of CAD and the computer-assisted subjective raters were all consistently high whereas the specificity of the CAD was lower than that of both subjective raters regardless of the TIRADS used.

## 2. Materials and Methods

### 2.1. Study Type and Data Sources

This retrospective study was approved by the Human Subjects Ethics Subcommittee of The Hong Kong Polytechnic University (Registration Number: HSEARS20190123004) and adhered to the Declaration of Helsinki guidelines. A consecutive case analysis approach was used for the data collection of thyroid nodule ultrasound images. Informed consent was waived for this retrospective study.

### 2.2. Image Selection Criteria

**Figure 1:**
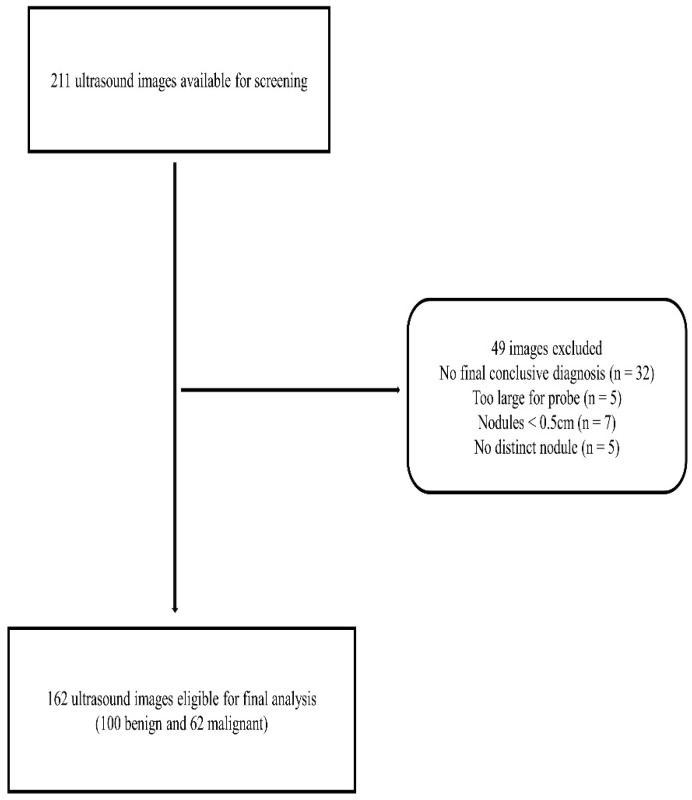
A flowchart showing the image selection criteria.

A total of 162 thyroid nodule ultrasound images were eligible for the analysis from the thyroid nodule images of patients that were prospectively scanned by our research group in the period between May 2019 and May 2021 (Figure 1). Standard thyroid ultrasound imaging protocols were observed to acquire the images using a Supersonic Aixplorer ultrasound machine (SuperSonic Imagine, Aix-en-Provence, France) and a 2–10 MHz linear transducer. A diagnostic radiographer with sonography experience and about 3 years of experience in thyroid ultrasound imaging solely performed the thyroid ultrasound scans. Ultrasound-guided fine-needle aspiration cytology (FNAC) was then independently conducted by two thyroid surgeons with extensive experience who later provided the cytological and/or histopathological diagnosis of the thyroid nodules.

The inclusion criteria were diagnostically acceptable thyroid nodule grey-scale ultrasound images from adult patients who had undergone ultrasound evaluation for thyroid cancer suspicion; and nodules ≥ 5 mm with complete size measurements in both transverse and longitudinal planes and taller-than-wide ratio assessment and confirmatory cytological and/or histopathological results. Ultrasound images of nodules that were too large for the ultrasound probe to completely show the lesion and patients without cytology or histopathology results were excluded from the study to not compromise the accuracy of the findings. Images of nodules < 5 mm were excluded because they were below the size criteria of the web-based malignancy risk assessment system. The reference standard was the conclusive FNAC and/or histopathology results of the nodules.

Transverse plane images demonstrating the most features suggestive of benignity or malignancy were selected and areas clearly demonstrating the nodule and its relationship to the adjacent thyroid parenchyma and surrounding structures were separated from the entire image. The new nodule-specific images were anonymized, coded, and saved in JPEG format.

### 2.3. Analyses of the Thyroid Nodule Images

The analyses were conducted separately with an online malignancy risk assessment system (http://www.gap.kr/xe/Estimation (accessed on 5 August 2021)) and AmCAD-UT version 2.2 (AmCad Biomed, Taipei, Taiwan) for the same nodules. The computer-assisted subjective analyses with the risk calculator were conducted first and the CAD analysis was performed after two weeks.

#### 2.3.1. Computer-Assisted Subjective Risk Assessment

**Figure 2:**
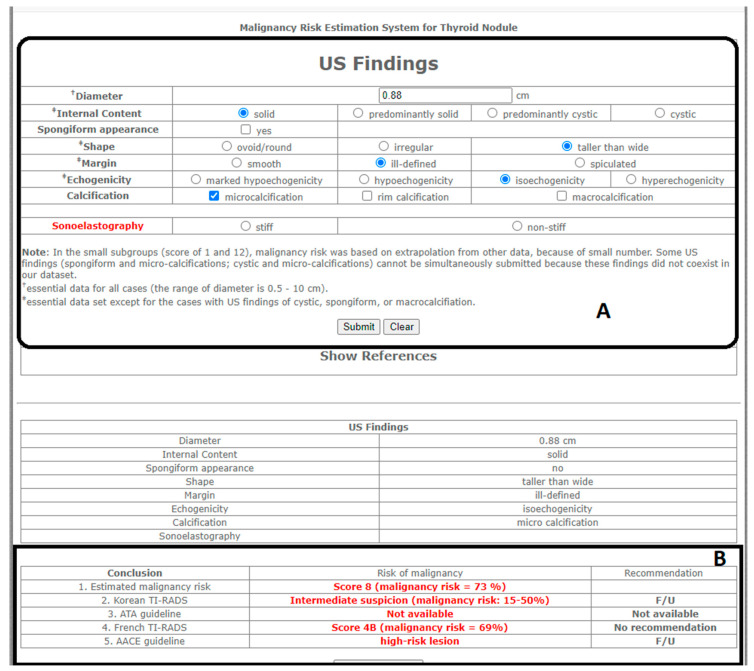
An image demonstrating the user interface of the web-based risk calculator. (**A**): The section for sonographic feature inputs based on an image observed by a subjective rater. (**B**): The malignancy risk stratification computed output obtained after the sonographic feature inputs have been submitted to the system. The output is based on AACE, ATA, KSThR, and French TIRADS.

Two raters independently reviewed the same set of ultrasound images and evaluated the ultrasound features of the thyroid nodules using the stipulated rating criteria of the online risk calculator (Figure 2A). Rater 1 (R_1_) was the radiographer who had performed all the thyroid scans. Since real-time image rating using the risk calculator was not practically feasible at the time of scanning, R_1_ rated the thyroid nodules simultaneously but independently, with another rater (Rater 2—R_2_) for comparison purposes. R_2_ was a senior sonographer, with over 15 years of experience, who was not involved in the imaging process and first encountered the ultrasound images during the rating process. Both raters were blinded to the cytology and histopathology results during their individual rating process.

The online calculator computed the malignancy risk based on a rater’s subjective assessment of the composition, margins, echogenicity, shape, and calcification from the images of each of the thyroid nodules [8]. In this study, a calculated taller-than-wide ratio of >1 was used to determine if a nodule was taller-than-wide in addition to subjective visual assessment [13,14]. The malignancy risk assessment was automatically computed as risk stratification category outputs for AACE, ATA, KTA/KSThR, French TIRADS, and an estimated malignancy risk (EMR) score (Figure 2B). In this study, the risk stratification outputs for the French TIRADS were converted to EU-TIRADS, an updated version of French TIRADS, based on the corresponding malignancy risk estimation percentages, for comparison with the AmCAD-UT EU-TIRADS outputs.

#### 2.3.2. CAD Assessment

**Figure 3:**
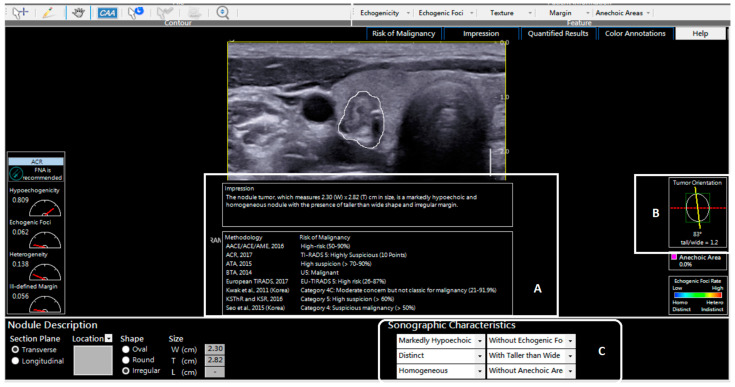
An example of a typical image analysis output from the thyroid CAD software. (**A**): The malignancy risk stratification of the nodule based on different classification systems. (**B**): The software’s computation of the taller-than-wide ratio. (**C**): The software’s computation of the sonographic features based on the analyzed image.

The coded JPEG thyroid nodule images were uploaded onto the AmCAD-UT software user interface for analysis. The AmCAD-UT algorithm allows for the selection of automated, semi-automated, or manual outline of the region of interest (ROI) for detecting the nodule for risk stratification. For this study, the default selection for outlining the ROI was the automated nodule segmentation with semi-automated manual correction when necessary. When the automatically segmented ROI demonstrated satisfactory nodule boundary outlines, the computed diagnosis was accepted as valid. When the automated nodule segmentation missed the nodule or under or over-estimated the nodule boundaries, manual correction was applied to the automated outline as determined by the more experienced rater of the two raters. Only 15 out of 162 nodules (9.3%) required manual correction of the automated ROI in the present study. The malignancy risk category output for each TIRADS, taller-than-wide ratio output, and sonographic characteristics outputs for each nodule on CAD (Figure 3) were compared to the corresponding entries for each computer-assisted rater. There was no output for the EMR score in CAD, and hence this was not compared between the different approaches.

### 2.4. Data Analysis and Statistical Analysis

All statistical analyses were performed using the SPSS software package (version 25.0, SPSS Inc., Chicago, IL, USA). Categorical variables were expressed as frequencies and continuous variables were expressed as mean values ± standard deviation. The Chi-square test was used to compare differences in classification data while the Mann Whitney U test was used to compare continuous variables. The Goodman and Kruskal’s Gamma correlation coefficient (G or γ) was used to measure the ordinal association of the sonographic features coded by the different raters. The gamma coefficient was interpreted as 0.01–0.30 negligible association, 0.31–0.50 low association, 0.51–0.7 moderate association, 0.71–0.9 high association, and 0.91–1.0 very high association [15]. The inter-rater reliability testing for the different raters was estimated using Cohen’s kappa statistic (κ). Proportions of agreement between paired ratings based on the different TIRADS category cut-off points for malignancy risk stratification was also used to determine absolute rater agreement. The Kappa result was interpreted as follows: 0.01–0.20 none to a slight agreement, 0.21–0.40 fair agreement, 0.41–0.60 moderate agreement, 0.61–0.80 substantial agreement, and 0.81–1.00 almost perfect agreement [16]. The sensitivity (SEN), specificity (SPEC), negative likelihood ratios (NLR), positive likelihood ratios (PLR), diagnostic odds ratio (DOR), and their corresponding 95% confidence intervals (CI) were calculated with reference to final cytology or pathology results. The McNemar and Cochran’s Q test for multiple comparisons were used for the comparative analysis of SEN, SPEC, and DA and post-hoc McNemar analyses were employed in the case of statistically significant results from the Cochran’s Q test. The differences in PPV and NPV were evaluated using a two-sample proportion test whereas the differences in LRs and DORs were evaluated based on 95% CIs, where non-overlapping values denoted statistical significance. The receiver operating characteristic (ROC) curves were generated to obtain the area under the ROC (AUROC) and the SPSS software computed the differences in AUROCs for paired comparisons. The optimal cut-off points for differentiating benign and malignant thyroid nodules were defined by the highest Youden’s J index based on the different categorizations of malignancy risk stratification for each of the 4 TIRADS used.

## 3. Results

### 3.1. Demographics and Thyroid Nodule Characteristics

This study included 162 thyroid nodules from 157 patients comprising 133 (84.7%) females and 24 (15.3%) males. The mean age of the patients was 53 years ± 13 (range 21–95 years). Of the thyroid nodules, 100 (61.7%) were benign while 62 (38.3%) were malignant; 41 (41%) of the benign nodules had benign cytology results whereas 27 (27%) had benign histopathology findings without defined pathology while the rest had pathological findings of goitre (n = 14), adenomas (n = 6), hyperplasia (n = 3), colloid nodule (n = 1), lymphocytic thyroiditis (n = 2), Hashimoto’s thyroiditis (n = 2), and Graves’ disease (n = 4). Of the malignant thyroid nodules, 54 (87%) were papillary thyroid carcinoma while the rest were follicular thyroid carcinoma (n = 5), Hurthle cell carcinoma (n = 1), and noninvasive follicular thyroid neoplasm with papillary-like nuclear features—NIFTP (n = 2). The mean diameter of all thyroid nodules was 1.57 ± 0.84 cm (range 0.5–3.9 cm). The mean diameter did not differ significantly between benign and malignant thyroid nodules (1.63 ± 0.85 cm and 1.46 ± 0.83 cm respectively, *p* = 0.15).

### 3.2. Nodule Sonographic Feature Classifications by Human Subjective Assessment and CAD

The different raters and the CAD system coded the sonographic features of the thyroid nodules based on echogenicity, calcifications, margins, composition, and shape. The results of the different categorizations are shown in Table 1. There were significant differences between the classifications of all sonographic features of benign and malignant nodules for both human raters (*p* < 0.05), whereas for the CAD system the significant differences were only observed for calcifications (*p* = 0.001). Amongst both human raters and CAD, majority of the malignant nodules were classified as hypoechoic compared to benign nodules (R_1_ = 66.1% vs. 48%, R_2_ = 72.6% vs. 49% and CAD = 48.4% vs. 34%). All raters classified the majority of the malignant nodules as either predominantly solid or solid (R_1_ = 16.1% and 79%, R_2_ = 54.8% and 41.9% and CAD = 38.7% and 61.3%, respectively). The classification of microcalcifications was predominant in malignant nodules when rated by R_1_ and CAD (46.8% and 51.6%, respectively), but with R_2_ malignant nodules were interpreted as mostly without calcifications or with microcalcifications (both 45.2%).

### 3.3. Classification Correlation Comparisons between Subjective Ratings and CAD

There was a high association in the rating of echogenicity of malignant nodules between both human raters and CAD (R_1_ vs. CAD, G = 0.74, and R_2_ vs. CAD_,_ G = 0.73), and a very high association between R_1_ and R_2_ (G = 0.91) as shown in Table 2. The human raters had a high association in stratifying calcifications and composition in all nodules and separate groups of malignant nodules and nodules (G > 0.7). There was negligible to a low association in classifying nodule margins between each of the human raters and the CAD for malignant, benign, and all total nodules (G < 0.5). The rank correlation association for categorizing the shape of benign nodules was generally high between each human rater and CAD and between the human raters (R_1_ vs. CAD, G = 0.81; R_2_ vs. CAD, G = 0.86; and R_1_ and R_2_, G = 0.85, respectively).

### 3.4. Rater Agreement Based on TIRADS

The rater agreement based on the malignancy cut-off points of the different TIRADS was generally moderate to substantial for malignant, benign, and all total nodules between the human raters (0.41 ≤ κ < 0.81), with the highest agreement achieved with ATA TIRADS (κ = 0.77). The results are shown in Table 3. The rater agreement between each of the human raters and the CAD was highest based on ATA TIRADS between R_1_ and CAD for all nodules (κ = 0.75), and lowest based on AACE for malignant nodules between R_1_ and CAD (κ = 0.12) and between R_2_ and CAD (κ = 0.14). There was a fair rate of agreement for classifying benign nodules with AACE (κ = 0.32) between R_1_ and CAD, and with ATA, EU, and KSThR (κ = 0.40, 0.24, and 0.23, respectively) between R_2_ and CAD for KSThR. Proportions of agreement between the different paired raters amongst all TIRADS were generally high in contrast to the moderate kappa values, although the agreement between R_2_ vs. CAD was low to moderate for benign nodules with all TIRADS—AACE = 50.8%, ATA = 73.7%, EU = 63%, and KSThR = 61% (Supplementary Table S3).

### 3.5. Diagnostic Performance Assessment of CAD and Computer-Assisted Raters for Matched TIRADS

The diagnostic performance outcomes for the two computer-assisted subjective raters and CAD were assessed for different TIRADS as outlined in Table 4. The best diagnostic performance for the different TIRADS was achieved at high risk (category 3) for AACE, high suspicion (category 5) for ATA and EU, and intermediate suspicion (category 4) for KSThR. EU and KSThR TIRADS were able to specify all nodules regardless of the rater, whereas AACE rating with CAD failed to specify some nodules (39 benign, 9 malignant) while ATA failed to specify some nodules regardless of the rater (CAD—30 benign, 19 malignant; R_1_—16 benign, 10 malignant; and R_2_—15 benign, malignant). Overall, the common nodules across all raters that could be specified by AACE, and ATA were 114 (61 benign, 53 malignant) and 96 (57 benign, 39 malignant), respectively.

Based on the different TIRADS, CAD yielded the highest sensitivity but lowest specificity and AUROC amongst all raters with AACE (92.5%, 26.2%, and 0.59, respectively) which were all statistically significant different from R_2_ (79.3%, 62.3%, and 0.72, *p* = 0.02 and <0.001). For stratifying all 162 nodules, R_1_ had overall higher diagnostic performance than CAD for all metrics for EU and KSThR. Although the differences were not statistically significant for EU, there was a statistically significant difference in AUROC for KSThR (0.67(95% CI: 0.59; 0.75) vs. 0.76 (95% CI: 0.68; 0.83, *p* = 0.02) (Supplementary Table S2). R_2_ had comparable sensitivity but higher specificity than CAD for KSThR (75.8% vs. 83.9%, and 61% vs. 46%, *p* = 0.02, respectively). Between the two computer-assisted subjective raters, there were statistically significant differences in sensitivity, but comparable specificity and AUROCs for both EU (85.5% vs. 71%, *p* = 0.04; 62% vs. 64% and 0.74 vs. 0.69, respectively) and KSThR (90.3% vs. 75.8%, *p* = 0.01; 51% vs. 61% and 0.76 vs. 0.74, respectively). Overall, CAD generally had lower PLRs, although these were comparable to those of the computer-assisted raters, while the lowest NLR was achieved with computer-assisted rating (KSThR—R_1_—0.19). The highest specificity and PLR across all raters was achieved with ATA with comparable sensitivity, specificity, and AUROC amongst all raters. At the best performance, the computer-assisted approach had higher DOR > 9 and higher AUROC > 0.7 than the CAD-based approach on all the TIRADS. Across all TIRADS, all raters yielded high sensitivity and high NPVs, but low—to—moderate SPEC, PPVs, and DAs (Supplementary Table S1).

## 4. Discussion

The results of the current study demonstrated that for matched pairs of risk-stratification systems, although the two approaches had comparable diagnostic performance, computer-assisted subjective interpretation using KSThR yielded a higher overall diagnostic accuracy than computer-aided diagnosis.

### 4.1. Interpretation of the Study Findings for Sonographic Feature Ratings between the CAD and Computer-Assisted Approaches 

The rank correlation associations of ratings of sonographic features were generally high between the computer-assisted subjective raters for echogenicity, calcifications, and composition and negligible for margin ratings. This implies that the two computer-assisted raters mainly varied in rating nodule margins. Margin characteristics are among the sonographic features highly predictive of malignancy. Therefore, the differences likely influenced the final malignancy-risk computation using the online calculator for AACE, EU, and KSThR TIRADS. Comparatively, for CAD vs. either subjective rater, moderate association existed mostly for echogenicity and shape. While the rater agreement was mostly moderate between R_1_ and R_2_, the comparable sensitivities and specificities reflect how the computer-assisted scoring approach accounts for diverse rating criteria in determining a risk category. 

The moderate correlation association between CAD and each of R_1_ and R_2_ and fair-to-moderate inter-rater assessment but with comparable sensitivities may be attributed to CAD’s reliance on textural and statistical feature analysis based on supervised machine learning [17,18]. While individual sonographic ratings may have been different, CAD outputs are influenced by the detected sonographic features within the automated or selected ROI. The sonographic features that are detected within an ROI depend on how a particular CAD algorithm was trained with different images for malignancy risk stratification. Therefore, while CAD interprets the same sonographic features that a subjective interpreter inputs for computation by a risk-calculator model, image quality can contribute to increased sensitivity in misinterpreted suspicious features with CAD. Contrarily, an experienced human assessor may still be able to accurately interpret an image with artefacts that CAD is sensitive to.

### 4.2. Interpretation of the Study’s Diagnostic Performance Outcomes

In the present study, two raters independently using an online-based risk calculator had similar sensitivity and good diagnostic accuracy based on AUROC, with higher specificity across all TIRADS than the CAD. However, statistically significant differences in specificity were only observed using KSThR and AACE. For all four TIRADS, the PLR were generally higher for the computer-assisted subjective raters than the CAD and the DOR was highest with R_1_ using any of the TIRADS (>9). For EU and KSThR, both R_1_ and R_2_ had comparable sensitivity with CAD; however, there were statistically significant differences between them. The implication of this is that CAD systems can be an objective second opinion resource in the event of ambiguity with subjective outputs. However, automated web-based risk systems with simultaneous output for multiple TIRADS may potentially overcome challenges with subjective ambiguity and the bias towards high sensitivity but low specificity of commercially-available CAD. Deep learning-based CAD approaches have been suggested to be more accurate and improve specificity; however, current studies on the commercially-available deep learning-based S-Detect 2 (Samsung Medison Co. Ltd., Seoul, Korea) still show low specificity [19,20,21,22]. AmCAD-UT also uses a deep-learning analysis approach for automated ROI selection and it similarly resulted in lower specificities than the computer-assisted approach in the present study. 

The comparable sensitivity but low specificity of commercial and non-commercial CAD systems to that of experienced clinicians has been established in previous studies [20,23,24,25]. However, a few studies have shown higher specificity with CAD in comparison with human examiners of variable experience [10,26]. A recent multi-center study on the CAD-based on KSThR yielded a good AUROC (0.75) with the highest sensitivity (90.5%) but lowest specificity (49.6%) than that of the radiologists regardless of their experience [11]. However, in the present study, the KSThR TIRADS had the highest AUROC with R_1_ and R_2_ (0.76 and 0.74, respectively) with the highest sensitivity achieved by computer-assisted rater R_1_ (SEN: 90.3%; SPEC: 51%) whereas CAD had a lower AUROC and specificity, but comparable sensitivity (0.67, 46%, and 83.9%, respectively). The multi-center study suggested that CAD KSThR be reserved for large cancer screening with subjective assistance supplemented by another TIRADS to increase specificity. However, this present study’s findings, more so, the lowest NLR of 0.19 (0.09; 0.42) by R_1_ using KSThR, suggest that the computer-assisted approach would be better than CAD for ruling out disease. Nonetheless, this present study had a smaller sample size and fewer raters, and hence there is a need for further validation studies.

Although ATA demonstrated good nodule discriminating ability (AUROC ≥ 0.7) overall for both approaches, this was achieved with 96 nodules due to a high rate of non-specified nodules using CAD (30.2% vs. R_1_ = 16% vs. R_2_ = 12.3%). Computer-assisted rating with AACE specified all nodules whereas CAD did not specify 29.6%, thereby suggesting the superior efficiency of the risk-calculator model for this TIRADS than the CAD.

### 4.3. Meaning of the Study and Implications

The computer-assisted subjective assessment approach had comparable diagnostic performance to that of the CAD approach for all the four TIRADS. However, the high sensitivity of the CAD is outweighed by a lower specificity, thereby resulting in a lower diagnostic accuracy than that of computer-assisted subjective interpretation using KSThR. Complementary to sensitivity and specificity outcomes, the DOR, PLR outcomes may aid the choice of TIRADS and approach to consider for clinical adoption as they are not prevalence-dependent [27,28]. With the odds of almost 10, for the best DOR compared to about 5 for CAD for rating all nodules, this suggests that computer-assisted rating is superior to CAD when using KSThR or EU for mainly detecting papillary thyroid carcinomas. However, both approaches have the potential for clinical diagnostic workflow adoption by non-radiologists experienced in thyroid ultrasound imaging for screening purposes in low-resource settings due to comparable high sensitivities and NPVs. Nevertheless, where parallel use of both approaches can be adopted based on the TIRADS that can stratify all nodules, the best choice for rule out purposes is likely computer-assisted subjective interpretation using KSThR. Either approach using EU may suffice for rule-in purposes. The use of ATA and AACE is probably best with computer-assisted subjective interpretation due to higher rates of non-specified nodules with CAD.

### 4.4. Limitations and Directions for Future Research

Limitations of this study include the retrospective nature of the selection of patients’ images with FNAC and/or histopathology results which cannot exclude selection bias. Secondly, the optimal cut-off points for the different TIRADS were derived from the data but not pre-determined and therefore require validation. Thirdly, the sample size was small with the malignant nodules being mostly papillary thyroid cancer, thereby limiting the generalization to the general population and other thyroid cancers. The prevalence of malignancy within this study (38%) may not be reflective of the actual prevalence in the general population. The value of real-time, subjectively-assisted CAD compared to retrospective automated CAD analysis and multiple computer-assisted raters of diverse experiences needs to be explored. Therefore, larger standardized prospective studies with a diverse representation of thyroid cancers and multiple raters are warranted to assess the validity and generalizability of the findings.

## Figures and Tables

**Table 1:** Classifications of sonographic features for differentiating benign and malignant nodules based on the ratings of the two raters and CAD.

Sonographic	R_1_			R_2_			CAD			*p*-Values		
Feature	M = 62	B = 100	T = 162	M = 62	B = 100	T= 162	M = 62	B = 100	T = 162	R_1_	R_2_	CAD
**Echogenicity**												
Isoechoic	9 (14.5%)	35 (35%)	44 (27.2%)	12 (19.4%)	45 (45%)	57 (35.2%)	11 (17.7%)	27 (27%)	38 (23.5%)	0.006	<0.001	0.13
Hyperechoic	5 (8.1%)	13 (13%)	18 (11.1%)	0 (0%)	5 (5%)	5 (3.1%)	13 (21%)	31 (31%)	44 (27.2%)			
Hypoechoic	41 (66.1%)	48 (48%)	89 (54.9%)	45 (72.6%)	49 (49%)	94 (58%)	30 (48.4%)	34 (34%)	64 (39.5%)			
M-hypoechoic	7 (11.3%)	4 (4%)	11 (6.8%)	5 (8.1%)	1 (1%)	6 (3.7%)	8 (12.9%)	8 (8%)	16 (9.9%)			
**Calcifications**												
None	18 (29%)	61 (61%)	79 (48.8%)	28 (45.2%)	78 (78%)	106 (65.4%)	27 (43.5%)	71 (71%)	98 (60.5%)	<0.001	<0.001	0.001
Macro-calc	2 (3.2%)	10 (10%)	12 (7.4%)	2 (3.2%)	2 (2%)	4 (2.5%)	0 (0%)	0 (0%)	0 (0%)			
Micro-calc	29 (46.8%)	25 (25%)	54 (33.3%)	28 (45.2%)	19 (19%)	47 (29%)	32 (51.6%)	23 (23%)	55 (34%)			
Rim calc	1 (1.6%)	1 (1%)	2 (1.2%)	0 (0%)	0 (0%)	0 (0%)	0 (0%)	0 (0%)	0 (0%)			
Mixed calc	12 (19.4%)	3 (3%)	15 (9.3%)	4 (6.5%)	1 (1%)	5 (3.1%)	3 (4.8%)	6 (6%)	9 (5.6%)			
**Margins**												
Well-defined	26 (41.9%)	71 (71%)	97 (59.9%)	31 (50%)	70 (70%)	101 (62.3%)	42 (67.7%)	80 (80%)	122 (75.3%)	0.001	0.001	0.093
Irregular	32 (51.6%)	26 (26%)	58 (35.8%)	18 (29%)	26 (26%)	44 (27.2%)	20 (32.3%)	20 (20%)	40 (24.7%)			
Microlobulated	0 (0%)	0 (0%)	0 (0%)	1 (1.6%)	2 (2%)	3 (1.9%)	0 (0%)	0 (0%)	0 (0%)			
Spiculated	4 (6.5%)	3 (3%)	7 (4.3%)	12 (19.4%)	2 (2%)	14 (8.6%)	0 (0%)	0 (0%)	0 (0%)			
**Composition**												
Spongiform	3 (4.8%)	15 (15%)	18 (11.1%)	1 (1.6%)	13 (13%)	14 (8.6%)	0 (0%)	1 (1%)	1 (0.6%)	0.005	0.014	0.9
Pred. cystic	0 (0%)	7 (7%)	7 (4.3%)	1 (1.6%)	7 (7%)	8 (4.9%)	0 (0%)	1 (1%)	1 (0.6%)			
Pred. solid	10 (16.1%)	23 (23%)	33 (20.4%)	34 (54.8%)	51 (51%)	85 (52.5%)	24 (38.7%)	35 (35%)	59 (36.4%)			
Solid	49 (79%)	55 (55%)	104 (64.2%)	26 (41.9%)	29 (29%)	55 (34%)	38 (61.3%)	63 (63%)	101 (62.3%)			
**Shape**												
Round/Ovoid	25 (40.3%)	74 (74%)	99 (61.1%)	28 (45.2%)	75 (75%)	103 (63.6%)	49 (79%)	90 (90%)	139 (85.8%)	<0.001	<0.001	0.057
Taller than wide	28 (45.2%)	20 (20%)	48 (29.6%)	27 (43.5%)	21 (21%)	48 (29.6%)	13 (21%)	9 (9%)	22 (13.6%)			
Irregular	9 (14.5%)	6 (6%)	15 (9.3%)	7 (11.3%)	4 (4%)	11 (6.8%)	0 (0%)	1 (1%)	1 (0.6%)			

M = malignant nodules, B = benign nodules, T = total nodules, M-hypoechoic = markedly hypoechoic, calc= calcifications, Pred. = predominantly, R_1_ = Rater 1, R_2_ = Rater 2, CAD = AMCAD-UT.

**Table 2:** Rank correlation (γ) of rating of sonographic features by the two raters and CAD.

Sonographic Feature	R_1_ vs. CAD			R_2_ vs. CAD			R_1_ vs. R_2_		
	M	B	T	M	B	T	M	B	T
Echogenicity	0.74	0.55	0.64	0.73	0.63	0.67	0.91	0.64	0.78
Calcifications	0.30	0.45	0.49	0.33	0.26	0.41	0.84	0.78	0.85
Margins	0.39	0.14	0.33	0.37	0.22	0.35	0.03	0.63	0.45
Composition	−0.25	0.46	0.28	0.15	0.60	0.46	0.81	0.79	0.83
Shape	0.65	0.81	0.76	0.40	0.86	0.71	0.41	0.85	0.72

M = malignant nodules, B = benign nodules, T = all total nodules, R_1_ = Rater1, R_2_ = Rater 2, CAD = AMCAD-UT.

**Table 3:** Rater agreement (κ) for the TIRADS classifications.

Raters	Nodules	TIRADS			
		AACE	ATA	EU	KSThR
R_1_ vs. CAD	M	0.12	0.69	0.23	0.43
	B	0.32	0.68	0.40	0.46
	ALL	0.35	0.75	0.46	0.53
R_2_ vs. CAD	M	0.18	0.57	0.45	0.45
	B	0.12	0.40	0.24	0.23
	ALL	0.21	0.56	0.38	0.37
R_1_ vs. R_2_	M	0.52	0.71	0.40	0.50
	B	0.60	0.71	0.57	0.43
	ALL	0.65	0.77	0.59	0.54

ATA = American Thyroid Association, AACE = American Association of Clinical Endocrinologists/American. College of Endocrinology/Associazione Medici Endocrinologi, EU—European Union, KSThR—Korean Society of Thyroid Radiology.

**Table 4:** Diagnostic performance metrics of the 2 raters and CAD based on different TIRADS.

Rater by TIRADS	N	SEN % (CI)	SPE (%) (CI)	PLR (CI)	NLR (CI)	DOR (CI)	AUROC (CI)
**EU-CAD**	162	79.0 (66.8; 88.3)	55.0 (44.7; 65.0)	1.76 (1.37; 2.26)	0.38 (0.23; 0.64)	4.61 (2.23; 9.53)	79.0 (66.8; 88.3)
**EU-R_1_**	162	85.5 (74.2; 93.1)	62.0 (51.8; 71.5)	2.25 (1.72; 2.95)	0.23 (0.12; 0.42)	9.61 (4.26; 21.68)	85.5 (74.2; 93.1)
**EU-R_2_**	162	71.0 (58.1; 81.8)	64.0 (53.8; 73.4)	1.97 (1.45; 2.68)	0.45 (0.30; 0.69)	4.35 (2.16; 8.37)	71.0 (58.1; 81.8)
**KSThR-CAD**	162	83.9 (72.3; 92.0)	46.0 (36.0; 56.3)	1.55 (1.26; 1.92)	0.35 (0.19; 0.64)	4.43 (2.03; 9.69)	83.9 (72.3; 92.0)
**KSThR-R_1_**	162	90.3 (80.1; 96.4)	51.0 (40.8; 61.4)	1.84 (1.49; 2.29)	0.19 (0.09; 0.42)	9.71 (3.84; 24.59)	90.3 (80.1; 96.4)
**KSThR-R_2_**	162	75.8 (63.3; 85.8)	61.0 (50.7; 70.6)	1.94 (1.47; 2.58)	0.40 (0.25; 0.63)	4.90 (2.42; 9.93)	75.8 (63.3; 85.8)
**AACE-CAD**	114	92.5 (81.8; 97.9)	26.2 (15.8; 39.1)	1.25 (1.06; 1.48)	0.29 (0.10; 0.81)	4.36 (1.35; 14.01)	92.5 (81.8; 97.9)
**AACE-R_1_**	114	88.7 (77.0; 95.7)	54.1 (40.9; 66.9)	1.93 (1.45; 2.58)	0.21 (0.10; 0.46)	9.23 (3.44; 24.79)	88.7 (77.0; 95.7)
**AACE-R_2_**	114	79.3 (65.9; 89.2)	62.3 (49.0; 74.4)	2.10 (1.48; 2.98)	0.33 (0.19; 0.58)	6.31 (2.72; 14.64)	79.3 (65.9; 89.2)
**ATA-CAD**	96	79.5 (63.5; 90.7)	66.7 (52.9; 78.6)	2.38 (1.60; 3.56)	0.31 (0.16; 0.59)	7.75 (2.99; 20.09)	79.5 (63.5; 90.7)
**ATA-R_1_**	96	79.5 (63.5; 90.7)	70.2 (56.6; 81.6)	2.67 (1.70; 4.19)	0.29 (0.15; 0.55)	9.12 (3.48; 23.87)	79.5 (63.5; 90.7)
**ATA-R_2_**	96	74.4 (57.9; 87.0)	68.4 (54.8; 80.1)	2.35 (1.54; 3.60)	0.37 (0.21; 0.66)	6.28 (2.53; 15.61)	74.4 (57.9; 87.0)

N = total nodules specified, SEN = sensitivity, SPEC = specificity, PLR = positive likelihood ratio, NLR = negative likelihood ratio, DOR = diagnostic odds ratio, AUROC = area under receiver operator. Characteristic curve, CI = 95% confidence interval.

## Data Availability

The clinical and ultrasound data are unavailable publicly due to patient confidentiality and privacy protection reasons.

## Supplementary Materials

The following are available online at https://www.mdpi.com/article/10.3390/life11111148/s1, Table S1: Prevalence-based diagnostic performance outcomes with sensitivity and specificity, Table S2: *p*- values for SEN, SPEC and AUROC comparisons between subjective raters and CAD per TIRADS.
