# Supplementary material for: Comparative Analysis of Computer-Aided Diagnosis and Computer-Assisted Subjective Assessment in Thyroid Ultrasound
Source: Life (Basel). 2021 Oct 27;11(11):1148. doi: 10.3390/life11111148 (PMC8621517; doi:10.3390/life11111148)
Supplement: Supplementary file 1 [file life-11-01148-s001.zip › life-1399539-supplementary.pdf]

Table S1: Prevalence-based diagnostic performance outcomes with sensitivity and specificity for common specified nodules across different TIRADS

RATER BY TIRADS	TOTAL NODULES	SEN % (CI)	SPE (%) (CI)	PPV (CI)	NPV (CI)	DA (CI)
**EU-CAD**	162	79.0 (66.8;88.3)	55.0 (44.7;65.0)	52.1 (41.6;62.5)	80.9 (69.5;89.4)	64.2 (56.3;71.6)
**EU-R$_1$**	162	85.5 (74.2;93.1)	62.0 (51.8;71.5)	58.2 (47.4;68.5)	87.3 (77.3;94.1)	71.0 (63.3;77.8)
**EU-R$_2$**	162	71.0 (58.1;81.8)	64.0 (53.8;73.4)	55.0 (43.5;66.2)	78.1 (67.6;86.4)	66.7 (58.8;73.9)
**KSThR-CAD**	162	83.9 (72.3;92.0)	46.0 (36.0;56.3)	49.1 (39.2;59.0)	82.1 (69.6;91.1)	60.5 (52.5;68.1)
**KSThR-R$_1$**	162	90.3 (80.1;96.4)	51.0 (40.8;61.4)	53.3 (43.3;63.1)	89.5 (78.5;96.0)	66.1 (58.2;73.3)
**KSThR-R$_2$**	162	75.8 (63.3;85.8)	61.0 (50.7;70.6)	54.7 (43.6;65.4)	80.3 (69.6;88.5)	66.7 (58.8;73.9)
**AACE-CAD**	114	92.5 (81.8;97.9)	26.2 (15.8;39.1)	52.1 (41.6;62.5)	80.0 (56.3;94.3)	57.0 (47.4;62.5)
**AACE-R1**	114	88.7 (77.0;95.7)	54.1 (40.9;66.9)	62.7 (50.7;73.6)	84.6 (69.5;94.1)	70.2 (60.9;78.4)
**AACE-R$_2$**	114	79.3 (65.9;89.2)	62.3 (49.0;74.4)	64.6 (51.8;76.1)	77.6 (63.4;88.3)	70.2 (60.9;78.4)
**ATA-CAD**	96	79.5 (63.5;90.7)	66.7 (52.9;78.6)	62.0 (47.2;75.4)	82.6 (68.6;92.2)	71.9 (61.8;80.6)
**ATA-R$_1$**	96	79.5 (63.5;90.7)	70.2 (56.6;81.6)	64.6 (49.5;77.8)	83.3 (69.8;92.5)	74.0 (64.0;82.4)
**ATA-R$_2$**	96	74.4 (57.9;87.0)	68.4 (54.8;80.1)	61.7 (46.4;75.5)	79.6 (65.7;89.8)	70.8 (60.7;79.7)

[a]

NPV = negative predictive value, PPV = positive predictive value, DA = diagnostic accuracy.

Table S2: p- values for SEN, SPEC, and AUROC comparisons between subjective raters and CAD per TIRADS

TIRADS	Diagnostic Performance Measure	Raters		
		R_1 vs CAD	R_2 vs CAD	R_1 vs R_2
**EU**	**SEN**	0.82	0.51	0.041
	**SPEC**	0.27	0.19	0.82
	**PPV**	0.40	0.70	0.67
	**NPV**	0.30	0.67	0.13
	**DA**	0.54	0.066	0.16
	**AUROC**	0.064	0.57	0.11
**KSThR**	**SEN**	0.57	0.31	0.010
	**SPEC**	0.21	0.020	0.37
	**PPV**	0.54	0.44	0.86
	**NPV**	0.26	0.79	0.15
	**DA**	0.74	0.70	0.23
	**AUROC**	0.021	0.13	0.41
**AACE**	**SEN**	0.50	0.017	0.089
	**SPEC**	0.001	<0.001	0.81
	**PPV**	0.17	0.12	0.81
	**NPV**	0.65	0.83	0.41
	**DA**	0.001	<0.001	0.30
	**AUROC**	0.001	<0.001	0.20
**ATA**	**SEN**	0.99	0.63	0.63
	**SPEC**	0.73	0.99	0.99
	**PPV**	0.79	0.98	0.77
	**NPV**	0.93	0.71	0.64
	**DA**	0.78	0.42	0.76
	**AUROC**	0.82	0.95	0.72

Table S3: Proportion of agreement (%) between raters based on TIRADS cut-off points for malignancy-risk stratification

Raters	Nodules	TIRADS			
		AACE (%)	ATA (%)	EU (%)	KSThR (%)
**R_1 vs CAD**	**M**	84.9	89.7	77.4	87.1
	**B**	63.9	87.7	71.0	73.0
	**ALL**	73.7	88.5	73.5	78.4
**R_2 vs CAD**	**M**	79.2	84.6	79.0	82.3
	**B**	50.8	73.7	63.0	61.0
	**ALL**	64.0	78.1	69.1	69.1
**R_1 vs R_2**	**M**	86.8	89.7	79.0	85.4
	**B**	80.3	87.7	80.0	72
	**ALL**	83.3	88.5	79.6	77.2
